# Supplementary material for: Human alveolar progenitors generate dual lineage bronchioalveolar organoids
Source: Commun Biol. 2022 Aug 25;5:875. doi: 10.1038/s42003-022-03828-5 (PMC9409623; doi:10.1038/s42003-022-03828-5)
Supplement: Supplementary file 2 — Supplementary Information [file 42003_2022_3828_MOESM2_ESM.pdf]

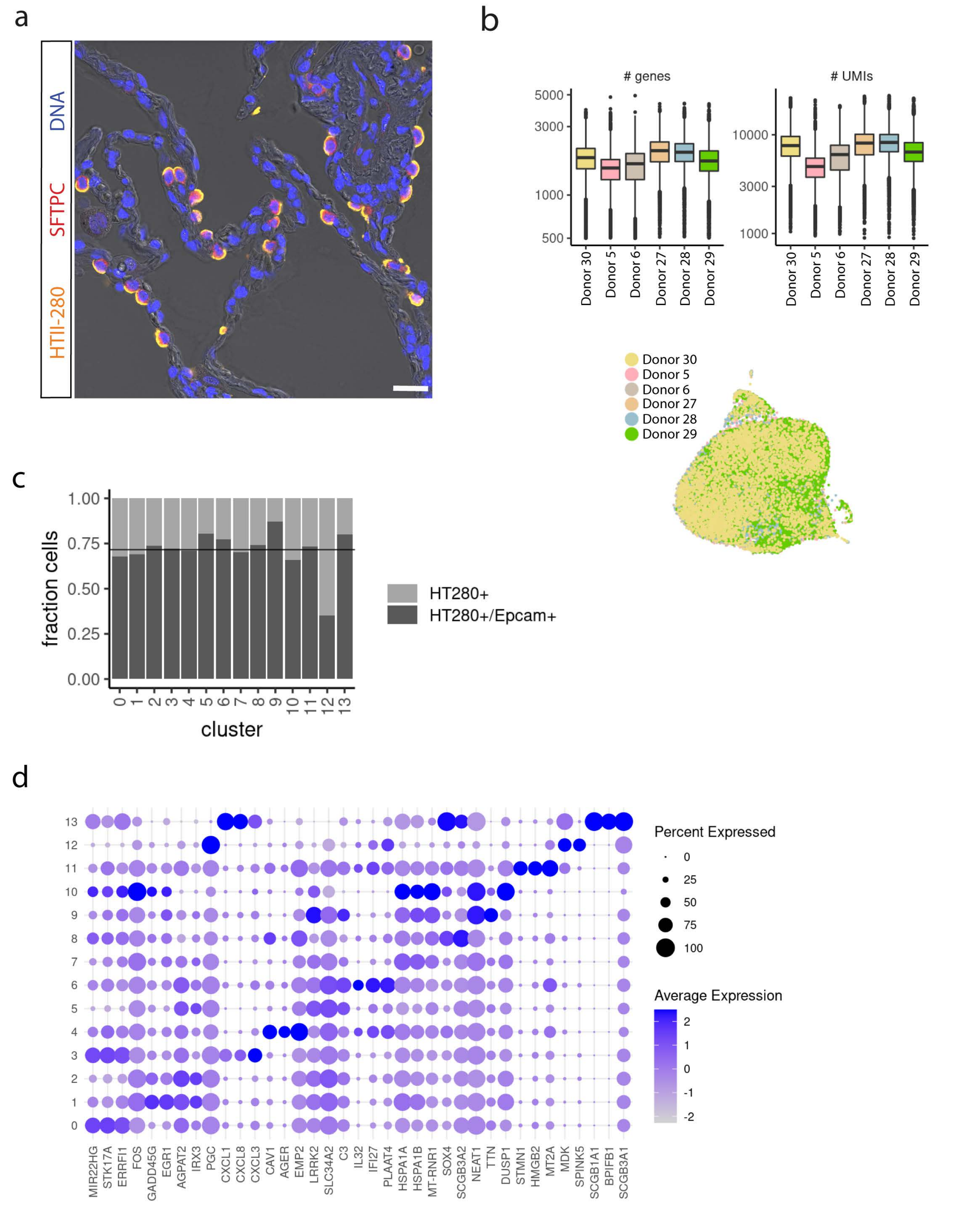

Supplementary Figure 1. HTII-280$^+$ cells in distal lung have AT2 phenotype.
Sup. Fig. 1 is related to Figure 1.
a Confocal image of HTII-280 and SFTPC in AT2 cells of peripheral human lung tissue. Scale bar: 20 µm. **b** Overview of the scRNA-seq metrics for the six donor tissues of HTII-280$^+$ cells and their distribution in UMAP embedding. **c** Overview of the fraction of HTII-280$^+$ and HTII-280$^+$/EpCAM$^+$ cells in each cluster. **d** Dot plot of cluster marker genes of HTII-280$^+$ cells.

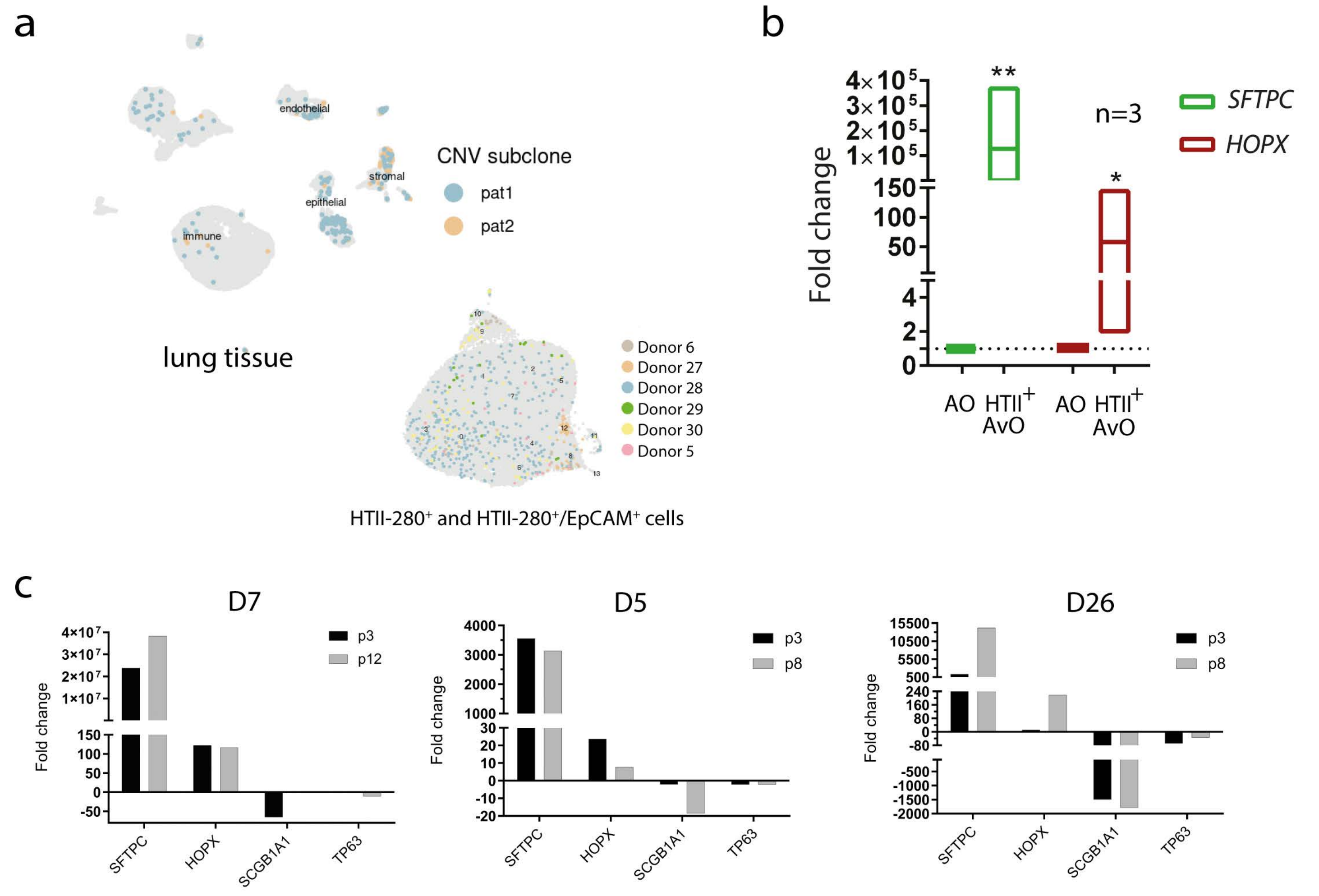

Supplementary Figure 2. Alveolar organoids maintain main differentiation markers in the long-term culture. Sup. Fig. 2 is related to Figure 2 and Figure 3.
a CNV analysis of scRNA-seq samples does not indicate presence of contaminating cancer cells.
b Validation of the alveolar marker induction by qPCR. n=3, $*p < 0.05$; $**p < 0.005$.
c Relative expression (qPCR) of differentiation markers during the long-term cultivation for 3 independent donor lines.

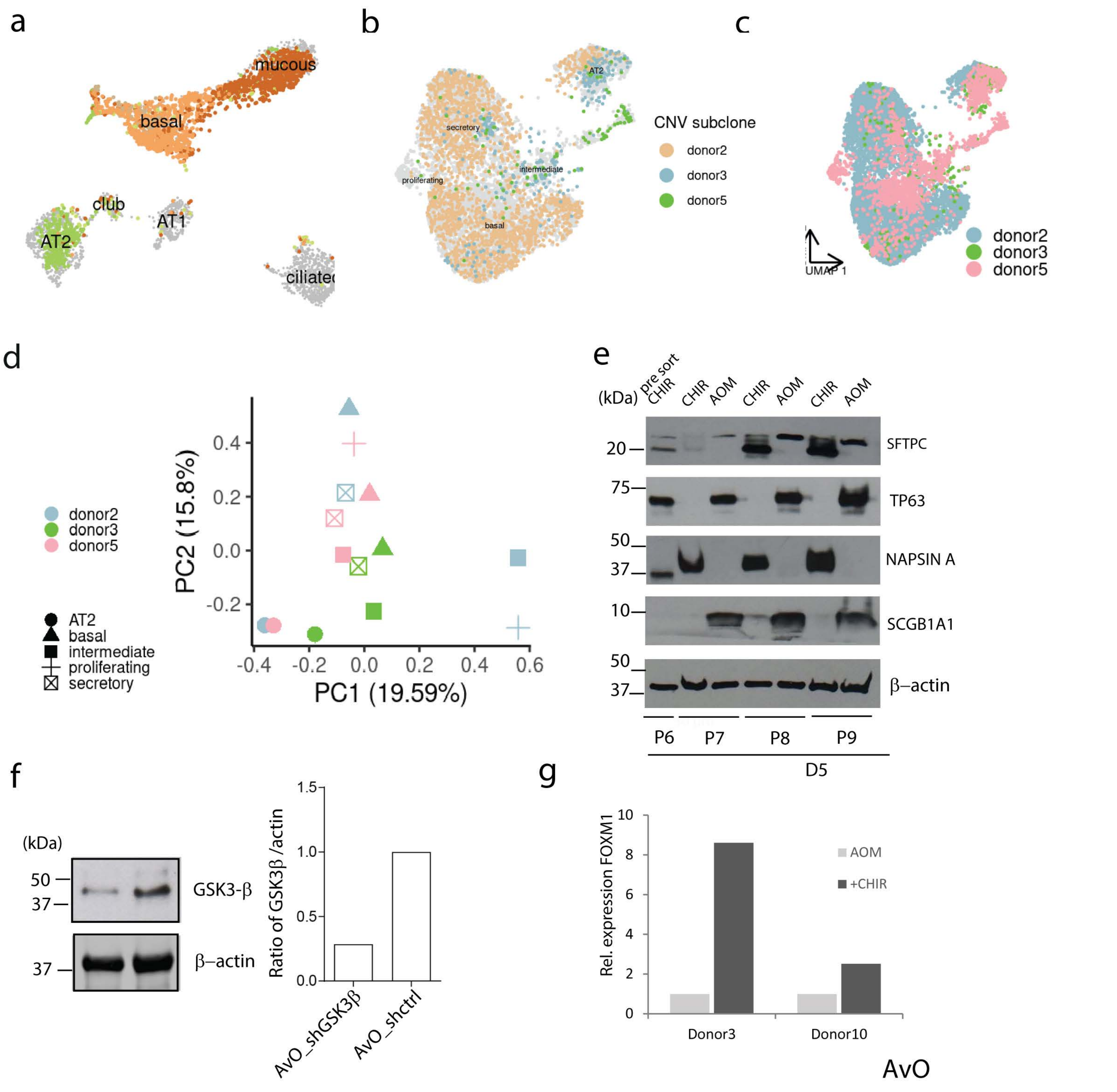

Supplementary Figure 3. HTII-280⁺ derived organoids in CHIR medium show sustained AT2 differentiation. Sup. Fig. 3 is related to Figure 3 and Figure 4.
a Joint embedding of HTII-280⁺-sorted CHIR-treated organoid cells (colored points) together with native lung epithelial cells (gray dots/labels) shows matching expression profiles. **b** CNV analysis of scRNA-seq of airway organoid samples does not indicate presence of contaminating cancer cells. **c** Distribution of cells from individual donors in HTII-280⁺ derived organoids. **d** Principal component analysis of average expression profiles for each donor of alveolar organoid lines. **e** Sub-sorted organoids grown in CHIR versus AOM medium over multiple passages show a stable and even increasing alveolar or airway phenotype, respectively, as indicated by high expression of SFTPC and NAPSIN in CHIR medium and TP63 and SCGB1A1 in AOM medium. f WB quantification by denistometry of the gsk3β knockdown **g** Validation of FOXM1 upregulation in CHIR-treated HTII-280-derived organoids compared to AOM from two different donors.

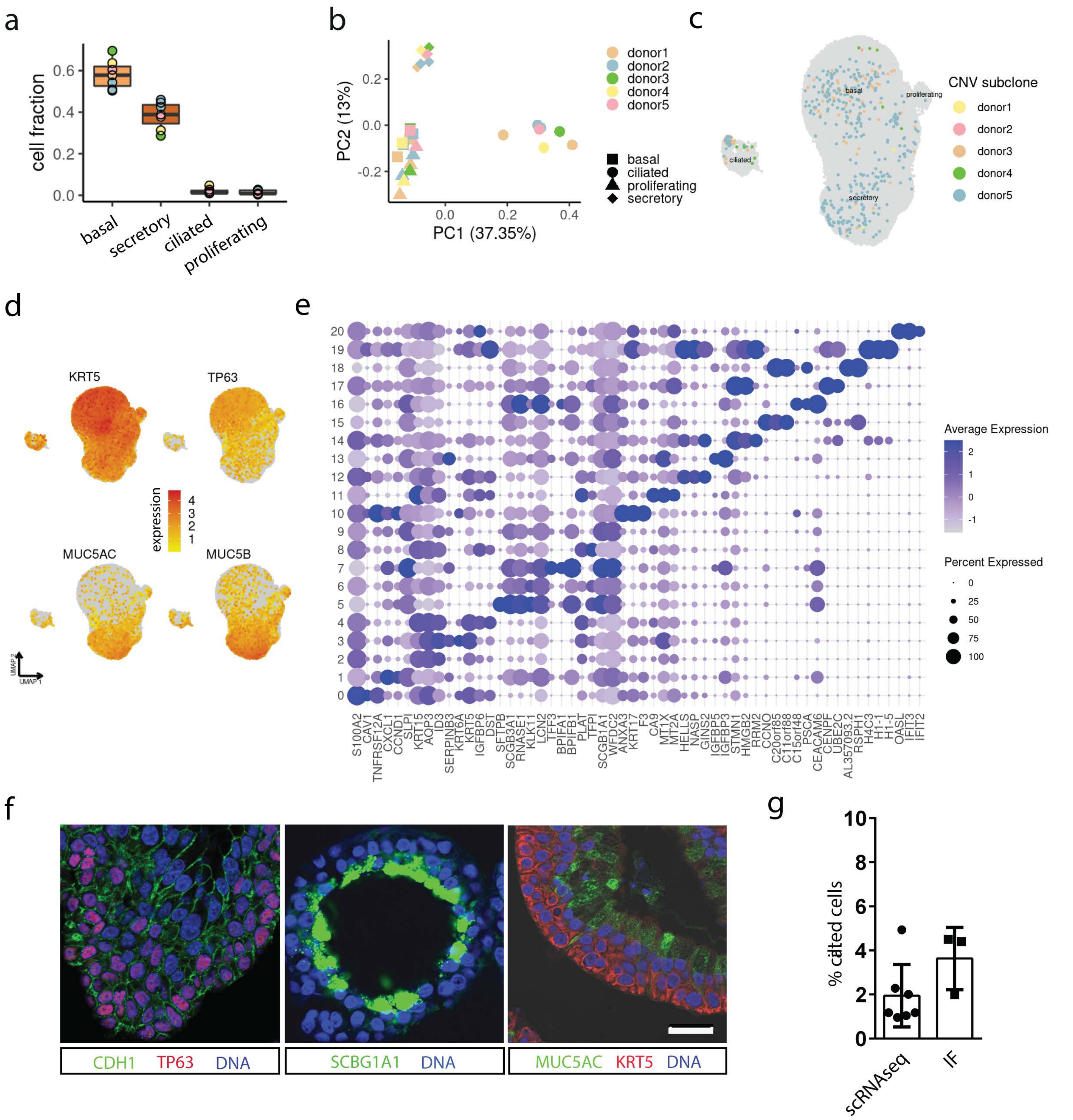

Supplementary Figure 4. Cell composition and differentiation phenotypes in airway organoids (AO) are the same across different donors. Sup. Fig. 4 is related to Figure 4 and 5. **a** A plot of cell-type proportions in pool airway organoids. **b** Principal component analysis of average expression profiles for each donor. **c** CNV analysis of scRNA-seq of AO samples does not indicate presence of contaminating cancer cells. **d** Selected marker genes of all identified subclusters color-coded for cell-type identities. **e** Dot plot of cluster marker genes for pool airway organoids. **f** Confocal images showing regions of the expanded basal layer (TP63, red, left) as well as differentiated club cells (SCGB1A1, green, middle) and secretory cells (MUC5AC, green, right). Scale bar: 20 µm. **g** Quantification of the fraction of ciliated cells with imaging (n=3) calculated as a percentage of ciliated cells per nuclei and by single-cell analysis (n=5).

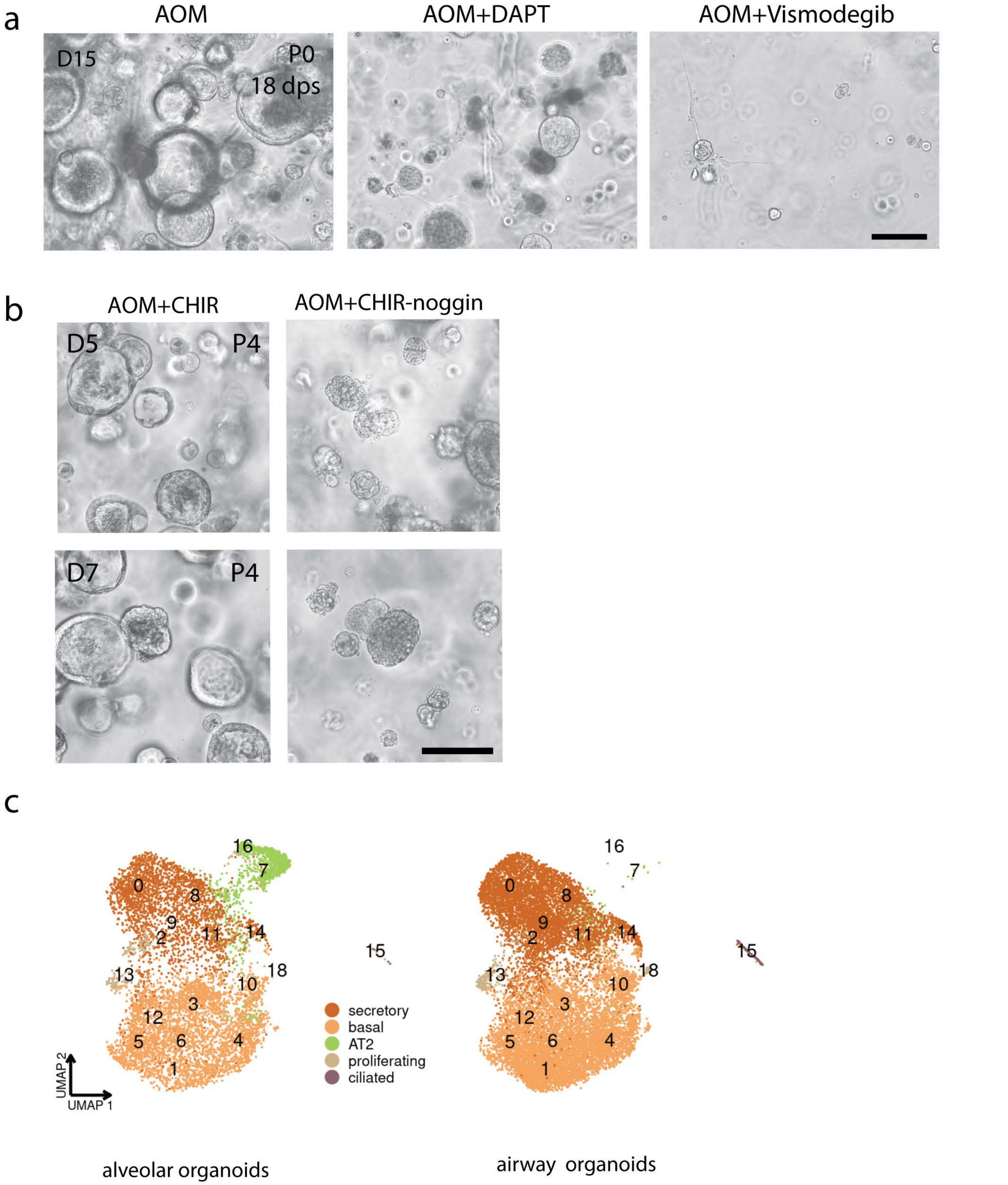

Supplementary Figure 5. SHH, NOTCH, and BMP signaling regulate organoid formation and growth. Sup. Fig. 5 is related to Figure 5 and 6.
a Representative phase-contrast images showing the negative effect of inhibition of SHH (Vismodegib) and NOTCH (DAPT) signaling on the formation of organoids. Scale bar: 100 µm. **b** Phase-contrast images from two independent donors showing the detrimental effect of noggin removal on the long-term expansion potential of the organoids. Scale bar: 100 µm. **c** Combined scRNA-seq analysis of alveolar and airway organoids derived from the same donors shows consistent cell type identification.

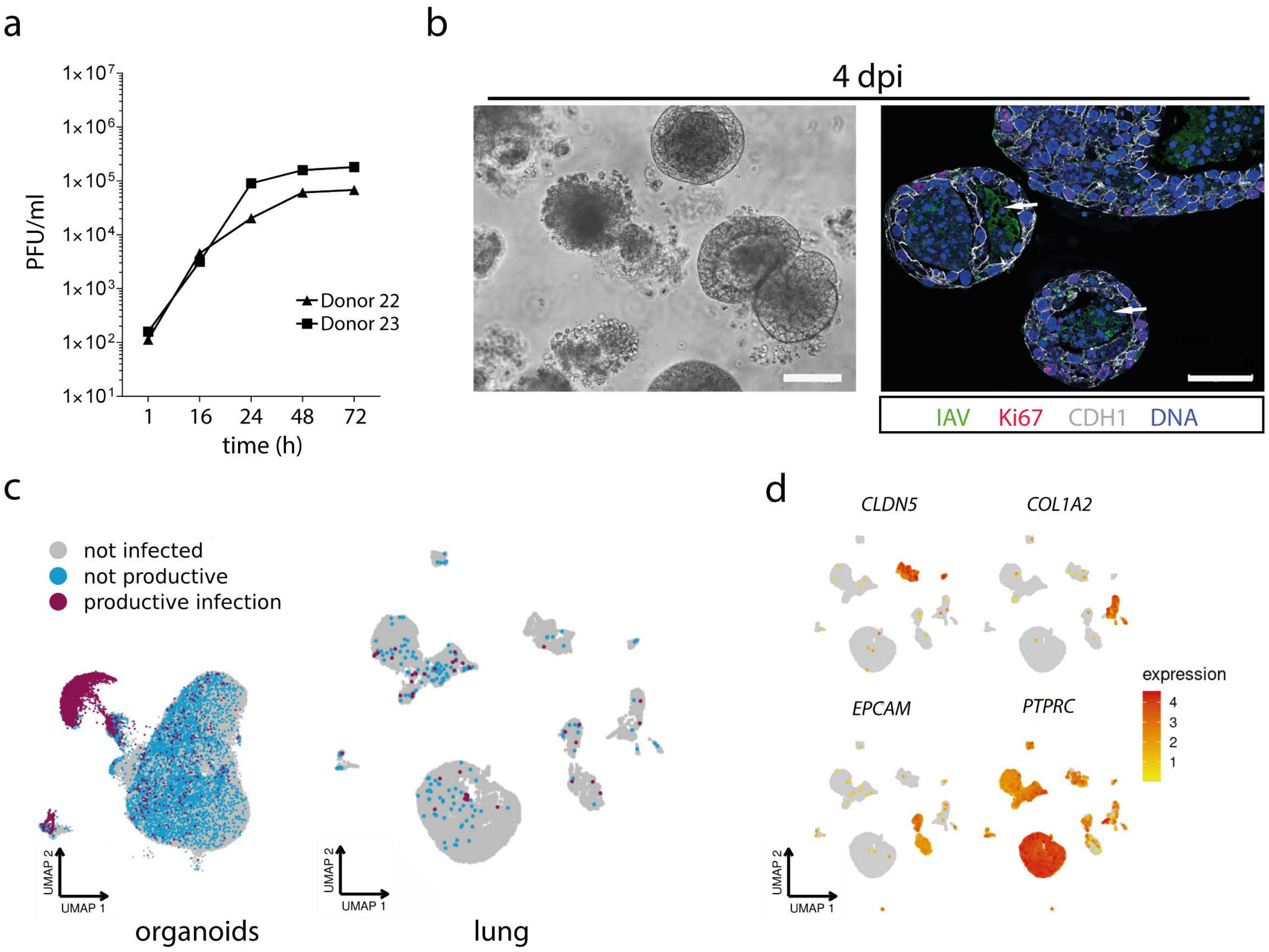

Supplementary Figure 6. Organoids survive IAV infection as infected cells are expelled to the lumen. Sup. Fig. 6 is related to Figure 7.
a Viral replication time course in two different ex vivo infected lung tissues.
b Phase-contrast (left) and confocal images (right) at 4 days post-infection (4dpi) show that organoids remain viable, with intact epithelial integrity while debris of infected cells is abundant in the lumen (arrows). Scale bars: 100 µm and 50 µm.
c Overview of likely productively infected, passively infected, and non-infected cells from organoids and ex vivo infected lung tissue in scRNA-seq UMAP plot.
d Distribution of main cell type marker genes in lung tissue fragments as analyzed by scRNA-seq.

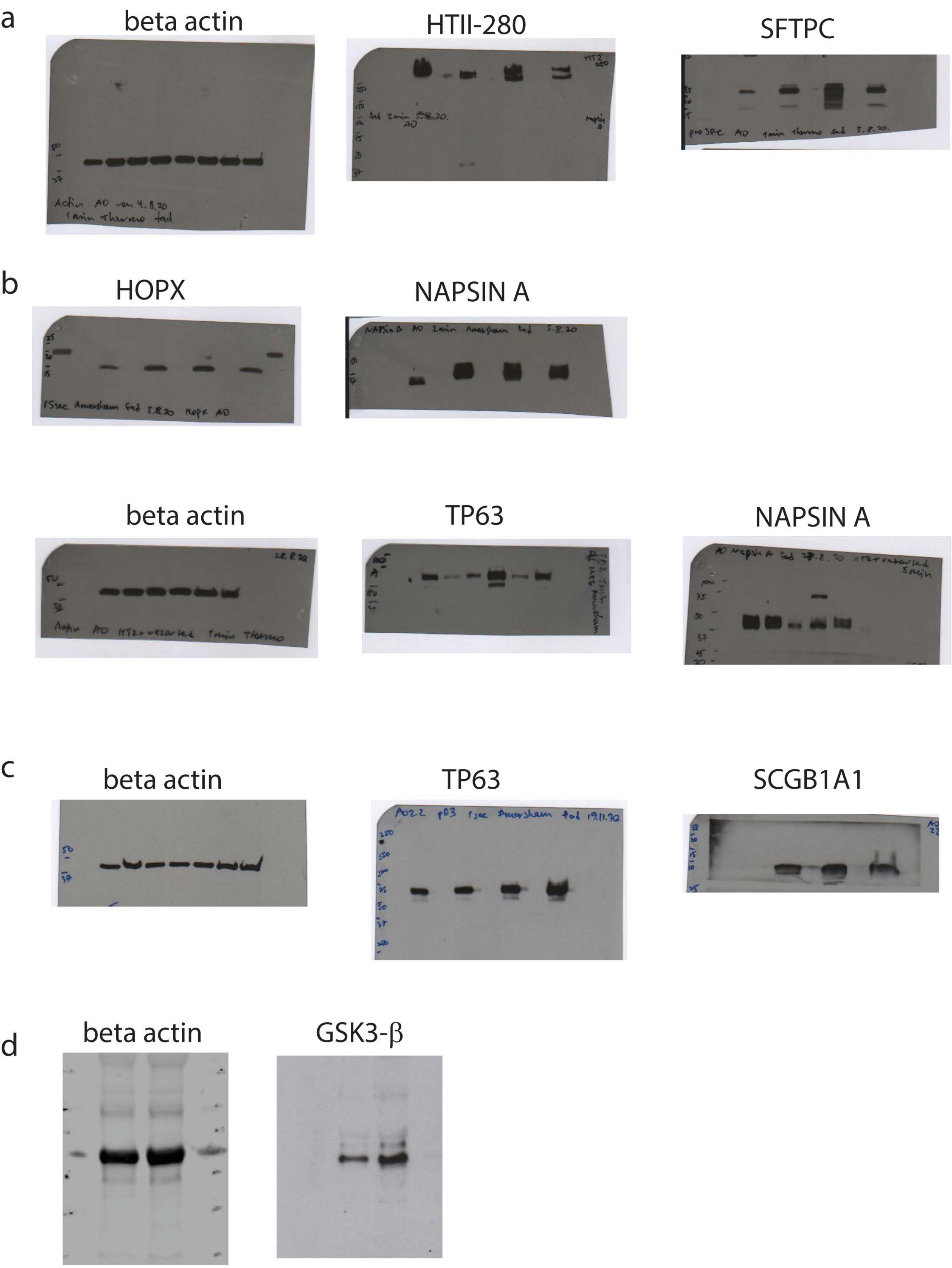

Supplementary Figure 7. Raw blots.
a Uncropped blots from Figure 3d. **b** Uncropped blots from Figure 4d. **c** Uncropped blots from Supplementary Figure 3e. **d** Uncropped blots of Supplementary Figure 3g.

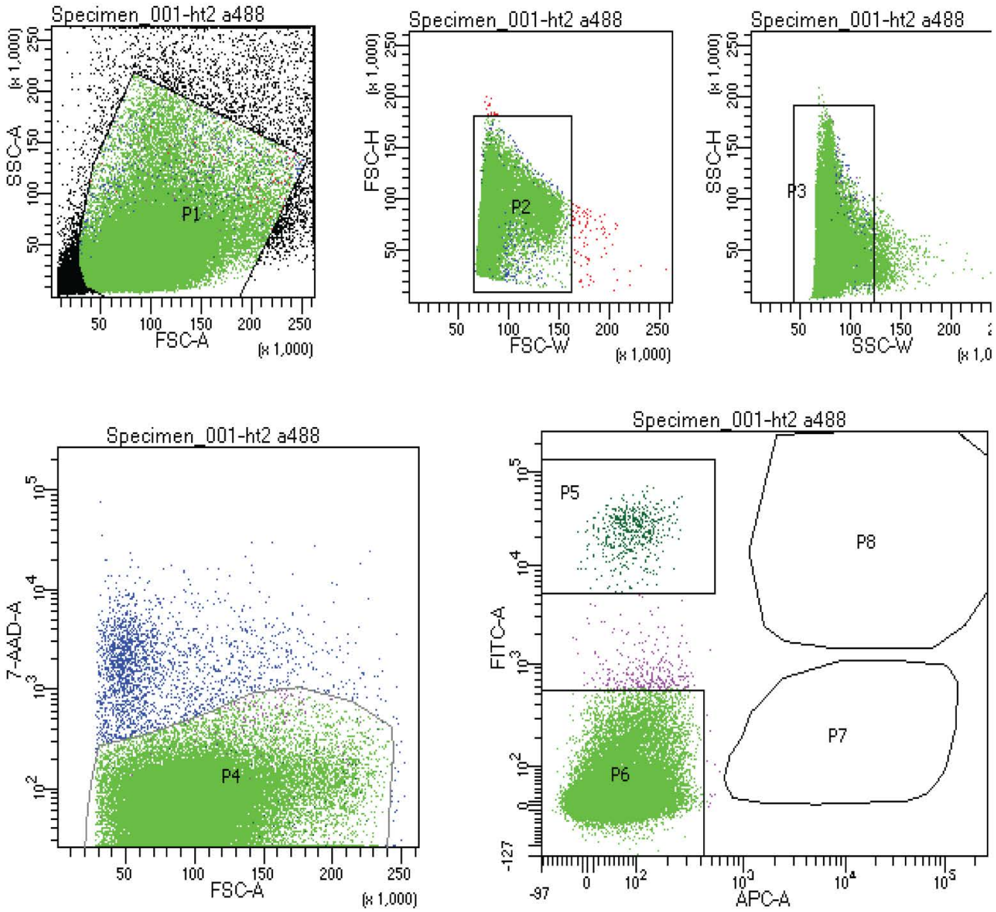

Tube: ht2 a488

Population	#Events	%Parent	%Total
All Events	80,016	####	100.0
P1	70,981	88.7	88.7
P2	70,874	99.8	88.6
P3	69,944	98.7	87.4
P4	67,663	96.7	84.6
P5	530	0.8	0.7
P6	66,836	98.8	83.5
P7	0	0.0	0.0
P8	0	0.0	0.0

Supplementary Figure 8. Gating strategy for sorting HTII-280^{+} cells from lung tissue.
