# Supplementary material for: Human alveolar progenitors generate dual lineage bronchioalveolar organoids
Source: Commun Biol. 2022 Aug 25;5:875. doi: 10.1038/s42003-022-03828-5 (PMC9409623; doi:10.1038/s42003-022-03828-5)
Supplement: Supplementary file 3 — Description of Additional Supplementary Files [file 42003_2022_3828_MOESM3_ESM.pdf]

Description of Additional Supplementary Files

File name: Supplementary Data 1

Description: List of marker genes, expressed in subclusters of HTII-280^{+} cells.

A list of adult stem cells Signature genes as identified by Smith et. al.

File name: Supplementary Data 2

Description: Lists of Differentially Expressed genes: in AOs AvO organoids, Differential gene expression in CHIR treatment Vs control and shGSK3b Vs Control, Differential gene expression during acute IAV infection.

File name: Supplementary Data 3

Description: Source data of the experiments underlying the graphs in the figures.
